# Relation of Childhood Home Environment to Cortical Thickness in Late Adolescence: Specificity of Experience and Timing

**DOI:** 10.1371/journal.pone.0138217

**Published:** 2015-10-28

**Authors:** Brian B. Avants, Daniel A. Hackman, Laura M. Betancourt, Gwendolyn M. Lawson, Hallam Hurt, Martha J. Farah

**Affiliations:** 1 Department of Radiology, University of Pennsylvania, Philadelphia, Pennsylvania, United States of America; 2 Department of Psychology, University of Pennsylvania, Philadelphia, Pennsylvania, United States of America; 3 Division of Neonatology, Children’s Hospital of Philadelphia, Philadelphia, Pennsylvania, United States of America; French National Centre for Scientific Research, FRANCE

## Abstract

What are the long-term effects of childhood experience on brain development? Research with animals shows that the quality of environmental stimulation and parental nurturance both play important roles in shaping lifelong brain structure and function. Human research has so far been limited to the effects of abnormal experience and pathological development. Using a unique longitudinal dataset of in-home measures of childhood experience at ages 4 and 8 and MRI acquired in late adolescence, we were able to relate normal variation in childhood experience to later life cortical thickness. Environmental stimulation at age 4 predicted cortical thickness in a set of automatically derived regions in temporal and prefrontal cortex. In contrast, age 8 experience was not predictive. Parental nurturance was not predictive at either age. This work reveals an association between childhood experience and later brain structure that is specific relative to aspects of experience, regions of brain, and timing.

## Introduction

How does childhood experience shape the brain? A large body of animal research shows that early experience affects later brain structure and function. Experimental manipulation of early life experiences has highlighted two especially influential aspects of early experience: the stimulation afforded by the environment, such as the variety of objects and opportunities for exploration and the presence of chronic stressors such as handling or long separations from the mother [1]. The latter influence is modulated by maternal behavior, which can buffer the developing brain from the effects of stress [2,3]. Although most of these studies have been carried out with rodents, similar findings have been obtained in nonhuman primates concerning environmental stimulation [4], infant stress [5] and maternal behavior [6].

It is not possible to test for similar effects of the environment on human brain development using equivalent experimental research designs. The most relevant evidence comes from observational studies in which naturally occurring variation in childhood experience is related to later measures of brain structure or function. Most such studies focus on extreme early experience such as the cognitive and social-emotional deprivation associated with early institutionalization [7,8] or maltreatment, neglect or the contrast between poverty and middle class environments [9–11]. Intervention studies with such populations offer some of the advantages of an experimental approach. However, such hardships bear an uncertain relation to normal healthy variation. It seems doubtful that physical assault or starvation by a parent or even the stresses of inadequate food, shelter and healthcare lies on a continuum with more or less sensitive, but adequate, species-typical parenting; in any case the relations between such extreme and normal experience is unknown. In addition, the global nature of the deprivation in such cases makes it impossible to separate the effects of cognitive deprivation from the effects of inadequate social-emotional parenting.

A complementary approach to assessing the effects of experience on human brain development comes from twin studies, which can partition the variance in brain structure across participants into variance associated with genetics and variance associated with the environments in which each pair of the twins has been reared (“shared environment”). Recent studies have assessed genetic and environmental contributions to whole brain, gray and white matter volume, white matter microstructure, cortical surface area and cortical thickness across many brain areas [12, 13]. These studies have estimated a substantial degree of heritability across many cortical regions. Consistent with the generally weaker power of twin studies to detect environmental influence [14], effects of shared environment are less often detected. A recent meta-analysis of twin studies derived reliable and often substantial estimates of genetic influence on cortical thickness across most of the different anatomically defined cortical regions measured, whereas influence of the shared environment, was generally much smaller than the influence of shared genes [12]. Unfortunately the influence of the nonshared environment, that is those aspects of environmental experience unique to an individual twin, is pooled with error in twin studies.

In addition to the separation of shared and nonshared environment, and the pooling of the latter with error, twin studies have other limitations as well for understanding the effects of environment on brain development. Like the studies of extreme variation, twin studies do not reveal the effects of specific aspects of the environment, for example quality of environmental stimulation and as distinct from quality of parental nurturance. Nor do they allow inferences about the timing of environmental influences, specifically the possibility that early childhood experience may be more influential on later life brain structure than later childhood.

The goal of the present research is to investigate the relationship between childhood experience and brain development with a new set of methods that will bring complementary strengths to those already available. Here we report the results of a first investigation of the relation between specific aspects of normal childhood experience and regional cortical thickness. Our study made use of a unique longitudinal dataset, from a sample of 52 healthy individuals whose childhood environments had been assessed by home visits at ages 4 and 8 and who then underwent high resolution structural MRI in late adolescence.

Three main questions are addressed by this study. The first two concern the selectivity of the relations linking these two different dimensions of early childhood experience and different regions of cortex: Rather than overall quality of childhood experience predicting later overall cortical thickness, do more specific relations hold?

Question 1, regarding regional brain structure, is: For which regions of cortex is thickness related to measured experience? Question 2, regarding the nature of the experience that is influential, is: Which aspect(s) of earlier childhood experience, Environmental Stimulation and/or Parental Nurturance, predict cortical thickness later in development? Question 3, concerning the timing of environmental influences and the question of a sensitive period, is: Does early childhood home experience exert more influence than later childhood home experience on adolescent cortical thickness? These questions were addressed by multiple regression relating childhood experience measures and covariates to regional cortical thickness.

## Materials and Methods

Fifty-two healthy young men (n = 25) and women (n = 27), average age 19.16 years (SD = 1.32) at the time of MRI, were recruited from a larger cohort followed since birth for a study of the effects of gestational cocaine exposure (13 GCE males, 12 control males, 14 GCE females and 13 control females) [15–18]. All were African American, born at term to low-income mothers with a mean gestational age of 38.4 weeks (SD = 2.2), a mean 5-minute Apgar score of 8.8 (SD = .6), normal cranial ultrasound and no asphyxiation. Mothers were native English speakers free of major psychiatric illness. None of the children had Fetal Alcohol Syndrome or any chromosomal disorder known to be associated with developmental delay. Since enrollment, the children have been evaluated semi-annually for measurements of growth, development, language, and cognitive and social-emotional outcomes [15–18].

Assent was obtained from all participating children and informed written consent was obtained from their parents or guardians. Subjects who were adults at the time of scanning signed written consent forms themselves. The project was conducted in accordance with the principles expressed in the Declaration of Helsinki and was approved by the Institutional Review Boards of the University of Pennsylvania and Children’s Hospital of Philadelphia.

Children’s home environments were evaluated at age 4 years (4.1 ± 0.2) and 8 years (8.4 ± 0.5) using the Home Observation for Measurement of the Environment (HOME) Inventory [19]. The HOME is a 1-hour structured interview and observational checklist that includes subscales measuring specific aspects of the child’s home life. Two composites, measuring environmental stimulation and parental nurturance, were created by averaging the z-scores of the relevant HOME subscales. The *Environmental Stimulation* composite incorporated subscales measuring opportunities for developing knowledge and skills (e.g., exposure to books, conversation, trips and music). The *Parental Nurturance* composite incorporated subscales measuring the warmth and availability of parental care (e.g., physical affection, verbal affection, discipline methods, parental involvement). For specific subscales and examples of items from each, see S2 File.

In addition to these composites, the following covariates were included in the analyses to be reported: gender, age at time of scan, the presence or absence of gestational cocaine exposure, the presence or absence of foster care, maternal Full Scale IQ (WAIS-III), and child Full Scale IQ (WAIS-IV), at age 18 years (and, in an alternate analysis, the verbal comprehension and perceptual reasoning indices from the WAIS-IV test at 18 years).

Scans were obtained from a Seimens 3.0 T Trio scanner with a 32-channel Siemens head coil, using a 3D MPRAGE sequence (TR: 1620 ms, TI: 950 ms, TE: 3 ms, flip angle: 15°, 160 contiguous slices of 1.0 mm thickness, FOV: 192x256 mm^2^, matrix: 192x256, 1NEX) and a total scan time of 5 min. Image quality was verified by visual inspection before further processing.

Analyses used publicly available and open-source Advanced Normalization Tools (ANTs, http://www.picsl.upenn.edu/ANTS/) and the associated pipelining framework PipeDream (sourceforge neuropipedream) using standard parameters for template construction, normalization, segmentation, and thickness estimation. For additional details see S1 File.

Regions of interest (ROIs) were defined across the entire cortex using the method of eigenanatomy [20–22], which clusters cortical regions with similar information into component vectors that are spatially coherent and ranks these regions by the variance they explain (S1 File). This enabled us to investigate the association of childhood experience with meaningful subdivisions of cortex and to improve detection power for potentially subtle effects by testing of ROIs that capture maximum variance. We extracted 30 sparse eigenanatomy components from the images.

The thickness of the cortex in regions corresponding to these components was submitted to a standard regression analysis examining the relationships between cortical thickness and the earlier home environment. Eigenanatomy regions were ranked in order of the amount of variance explained and then tested, in that order, for relations with Environmental Stimulation, Parental Nurturance and covariates using multiple linear regression (R 2.15.1), with ages 4 and age 8 measures analyzed separately. Note that the testing order of eigenanatomy ROIs is statistically independent of our variables.

## Results

In answer to the first question, concerning selectivity of the relations linking childhood environment and later brain structure, a subset of the regions showed a significant relation. We tested the top 30 eigenvectors (explaining > 98% of the variance in cortical thickness). A total of 4 eigenanatomy regions (the 5th, 12th, 15th and 18th eigenanatomy components) were found to have a significant relationship with our childhood experience variables (q < 0.05) where a q-value represents a false discovery rate (FDR) corrected p-value. Table 1 shows model results including beta weights and corrected q-values for the predictive HOME variable, age 4 Environmental Stimulation, and the identity and significance levels for all other factors that were predictive within each area’s regression model. In all regions, more Environmental Stimulation predicted thinner cortex. Covariates listed in the table include gestational cocaine exposure, associated with thicker cortex (significant for two areas at the p < 0.05 level, uncorrected), age at scan, with older age associated with thinner cortex (significant for two areas at the p < 0.05 level, uncorrected), and gender, with female gender associated with thinner cortex (significant for two areas at the p < 0.01 and p<0.001 level, uncorrected). Notably, none of the four areas showed significant relations with the subjects’ IQ, maternal IQ, or foster care. To explore the possibility that more specific components of the subjects’ IQ might predict cortical thickness in these areas where full scale IQ does not, or might account for variance now attributed to Environmental Stimulation, we repeated the analysis with the WAIS IV Verbal Comprehension Index and Perceptual Reasoning Index in lieu of the Full Scale IQ. These are the two components of the WAIS General Ability Index, comprising the Similarities, Vocabulary and Information subtests and Block Design, Matrix Reasoning and Visual Puzzles subtests, respectively. Neither index was predictive of cortical thickness in these areas, and their inclusion did not change the pattern of results obtained for the other variables of interest. Numerical detail regarding these analyses is reported in S2 File.

**Table 1:** Cortical eigenanatomy related to environmental stimulation in the home at age 4. Brodmann Areas are determined via a template-based mapping between our population-specific coordinate system and the standard Talairach space. The primarily left inferior temporal gyrus eigenanatomy region includes a contribution from prefrontal cortex; the primarily right inferior temporal gyrus includes a small contribution from contralateral angular gyrus. Model beta values and standard error for age 4 environmental stimulation are also shown. The middle columns show uncorrected **p**-values and FDR-corrected **q**-values for the relation of cortical thickness to environmental stimulation. Other variables entered into the models were age at scan, gender, gestational cocaine exposure (GCE; yes or no), history of foster care (yes or no), parental nurturance composite from the HOME, maternal IQ and child IQ (at age 18). The right-most column shows other factors in the model that were related to cortical thickness with at least p<0.05 significance, uncorrected.

Principal brain regions				Environmental stimulation, age 4			Other significant factors
Brief Descriptio of Eigen-anatomy	Brodmann Areas	Talairach Coords	Fig 1 Color	Standardized Beta	p	q	Level of significance * p<0.05, ** p<0.01, ***p<0.001
L lateral inferior temporal—posterior	20	5, -11, -27	yellow	-0.479	0.002	0. 047	GCE* (control Ss thinner), age* (older Ss thinner)
L lateral Inferior temporal -anterior	20	54, -11, -27	blue	-0.599	0.000	0.010	GCE* (control Ss thinner), age* (older Ss thinner)
Bilateral fusiform	37	+/-42, -58, -10	violet	-0.433	0.000	0.047	Gender*** (Female Ss thinner)
R lateral inferior temporal	20	-61–29–14	red	-0.448	0.005	0.047	Gender** (Female Ss thinner)

**Fig 1:**
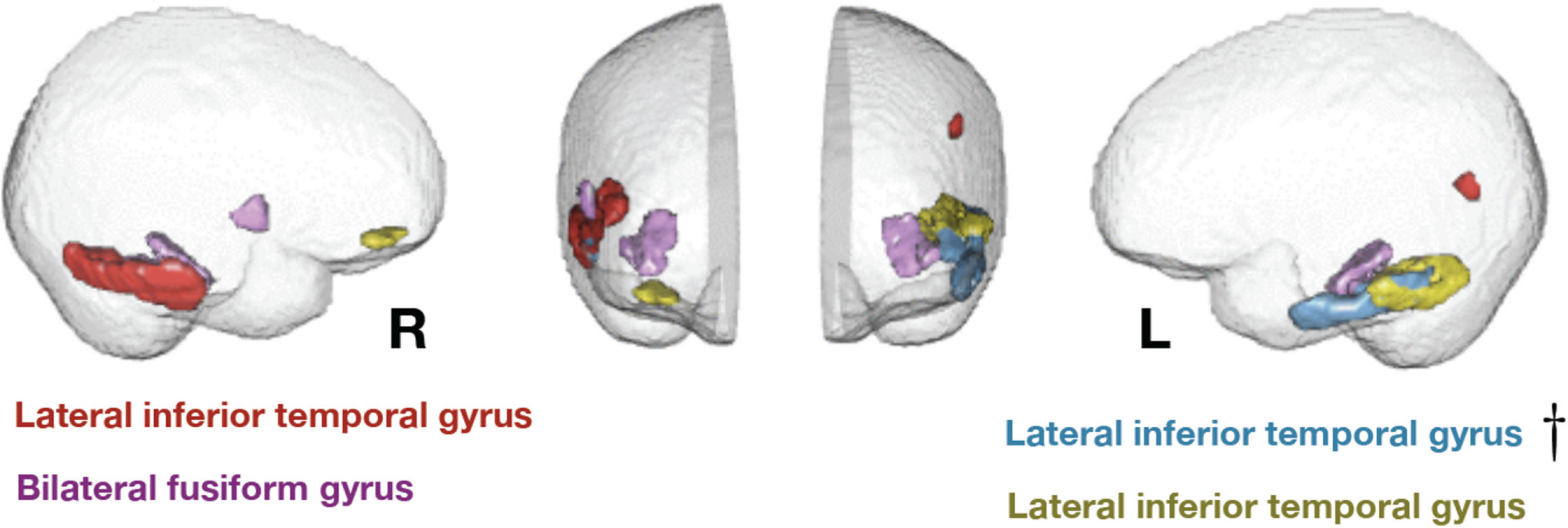
Eigenanatomy regions where cortical thickness is significantly predicted by age 4 environmental stimulation. Effects are present in bilateral fusiform and lateral inferior temporal lobe. Two components show a small amount of network structure with some frontal involvement on the right side and some parietal involvement on the left. The most significant region, in Brodmann area 20 on the left, is denoted by a dagger and survives family-wise error rate correction.

Parental Nurturance also failed to predict thickness in these four, or any other, regions. These regions are shown in Fig 1. They included both hemispheres, mainly in lateral and inferior temporal cortex but also including a right prefrontal region.

Addressing the second question, selectivity in terms of the aspects of experience that are predictive, selectivity was again observed. For all four regions, Environmental Stimulation was predictive and Parental Nurturance was not. Controlling for family-wise error reduces the number of related regions to the single most significant region found by FDR correction, in left inferolateral temporal cortex, situated mid-way along the temporal lobe. Brodmann areas and Talairach coordinates for each region are shown in Table 1. These regions are predominantly associated with cognitive and multimodal (as opposed to perceptual, motor, or affective) processing.

**Fig 2:**
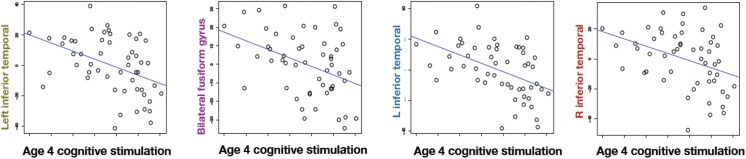
Increased environmental stimulation in the home at age 4 decreases cortical thickness as measured during young adulthood. The scatter plots show environmental stimulation in the home environment versus residualized cortical thickness in each of the significant regions. The thickness is residualized against the nuisance covariates of age at scan, maternal IQ, gender, foster care and prenatal cocaine exposure before plotting.

Fig 2 shows environmental stimulation at age 4 plotted against residualized cortical thickness in the four regions that survive FDR correction. To avoid circularity, we do not report inferential statistics on these areas analyzed separately because they were selected for their strength of relation [23]. The same regression performed with age 8 measures of Environmental Stimulation and Parental Nurturance failed to reveal any significant or trend relations with any of the first 30 eigenanatomy examined (all q’s > 0.8).

To address the third question posed earlier, that of a sensitive period, the differential predictive power of Environmental Stimulation at ages 4 and 8 was tested. A hierarchical regression was carried out, predicting cortical thickness in the four eigenanatomy regions as before, with age 8 Environmental Stimulation entered into the regression model along with all covariates, followed by Environmental Stimulation at age 4. The addition of the age 4 Environmental Stimulation variable significantly improved model fit to predict thickness in the posterior lateral inferior temporal gyrus (ΔF (1,45) = 10.47, p = .002, ΔR^2^ = .150), anterior lateral inferior temporal gyrus (ΔF (1,45) = 14.91, p < .001, ΔR^2^ = .199), bilateral fusiform gyrus (ΔF (1,45) = 14.72, p < .001, ΔR^2^ = .170), and right inferior temporal gyrus (ΔF (1,45) = 8.25, p = .006, ΔR^2^ = .113) regions.

## Discussion

The effect of early life experience on later brain structure is a central topic in neuroscience, but difficult to study in humans. Studies relating variation in childhood experience to brain structure have focused on extreme adversity, such as institutionalization, abuse and neglect. While it is vitally important to understand the effects of adversity on brain development, these experiences do not represent normal variation in childhood experience. They are also uninformative regarding the distinct contributions of environmental stimulation and parental nurturance to brain development, given the globally abnormal nature of the experiences.

Twin studies illuminate the effects of normal experience, but are more effective at identifying genetic than environmental influences, as they can only estimate the effects of twins’ shared environment as distinct from measurement error and do so with generally lower power. As with studies of adversity, twin studies do not distinguish the effects of different aspects of the environment, such as the predominantly cognitive dimension of environmental stimulation and the predominantly affective dimension of parental nurturance assessed here.

The present study, by relating prospectively measured childhood experience to late adolescent brain structure, offers a first direct look at the relation between normal variation in childhood experience and later brain structure. This new approach to understanding the biological embedding of experience is not without weaknesses, but offers different strengths and weaknesses to previous approaches.

On the basis of these strengths of the present research design we were able to discern specificity in the relation of childhood experience to later brain structure. For the cortical thickness measures used here, environmental stimulation was predictive, independent of parental nurturance. Parental nurturance is indisputably important for brain development. Although, the HOME-derived measures of experience failed to reveal an effect of normal variation in parental nurturance on cortical thickness, it is possible that other measures of parental nurturance or other measures of brain development might have been related.

In addition, there was specificity in the timing of influence, with earlier environmental stimulation predicting later cortical thickness over and above later environmental stimulation. As with the lack of a parental nurturance effect on cortical thickness, or for that matter any other null result for a factor in regression analysis, the lack of influence of later measures of home experience does not mean that future studies could not find later effects. Indeed learning is known to affect brain structure in adulthood [24]. Here we wish to emphasize simply that whatever effects of age 8 home experience might be present in the teen brain, they were not detectable here whereas the age 4 effects were.

Finally, anatomical specificity was also observed. The cortical regions that relate to age 4 environmental stimulation included a small region in right inferior prefrontal cortex and several regions within inferolateral and inferior temporal cortex bilaterally. The most significant relation, with left lateral inferior temporal cortex, survives FWE correction as well as the FDR correction applied to all results reported. In all cases, more environmental stimulation at age 4 was associated with thinner cortex over a decade later.

Is thinner cortex is a sign of better or worse development in the areas affected by childhood environmental stimulation? One approach to answering this question would be to determine whether thinner cortex is associated with higher or lower IQ in the study participants. The lack of an association found here between cortical thickness and either full scale IQ, verbal IQ or performance IQ fails to support either hypothesis.

Evidence from other research is partly consistent with the idea that the thinner cortex associated here with more environmental stimulation reflects better development. In most parts of the cortex, from school years through young adulthood, there is a trend of thinning over time [25–27], consistent with the view that thinner cortex can be viewed a mark of more advanced development. Also consistent with this is evidence from some developmental disorders, associated with delayed thinning (in the case of Attention Deficit Hyperactivity Disorder [28]) or thicker cortex (in the case of autism [29]). Less consistent with a “thinner is better” view is the finding of a generally positive relation between intelligence and cortical thickness across a wide range of ages [30, 31]. However, the relation between IQ and cortical thickness is negative at the youngest ages examined [26], which may be the most relevant to the present findings. To understand why, given the older subjects studied here, note that published findings from older subjects do not analyze for or stratify by early life characteristics, in this case IQ. Such studies therefore reveal brain-behavior relations between contemporaneous behavioral and anatomical characteristics, rather than the long-range relations between early childhood and late adolescent brain structure discussed here.

As for the microanatomical basis of the present findings, it is tempting to interpret them in terms of pruning of cells and connections that occurred in early childhood, but this is conjectural. Differences in the genesis of cells and synapses, as well as differences in myelination (which can affect the appearance of the border between gray and white matter and hence the measured cortical thickness) are all possibilities as well [32].

Although the present findings indicate that early childhood home experience is more influential than later, precise inferences regarding timing cannot be made. In the absence of HOME measurements at ages other than 4 and 8, we cannot know the specific times at which the home environment is most influential. It is unlikely that age 4 represents a unique time of environmental sensitivity. Rather, the age 4 measures are presumably more correlated with measures from the maximally sensitive period, perhaps toddlerhood, than are the age 8 measures. One possible explanation for the changing sensitivity of cortical thickness to the home across the two ages studied here is that children spend relatively more time outside the home at age 8 compared to age 4, thus reducing the opportunities for influence by the home environment. If so, it would remain true that home environment is more influential in earlier than later childhood, but for reasons that are social rather than intrinsically biological.

Another limitation of the present study concerns the representativeness of the sample. Our participants were from African American families of low socioeconomic status, which contrasts with the typically higher SES samples of predominantly White participants used in most published brain imaging studies. One difference between our low SES sample and the equally narrow SES ranges of higher SES samples is that low SES samples are likely to exemplify a broader range of environmental quality [33, 34]. This may increase the sensitivity of observational research designed to find correlates of environmental quality. There is no reason to believe that the relations between childhood experience and later cortical thickness would differ between racial or ethnic groups, but nevertheless the generality of the present findings cannot be known with certainty. The sample is also nonrepresentative in that half of the participants have a history of gestational cocaine exposure. In contrast to early expectations that so-called “crack babies” would be seriously impaired, the current medical consensus is that individuals born at term (such as those in the present study) have normal physical and mental health [35]. To take subtle residual effects of GCE into account in the present study, GCE was included as a covariate in the present analyses.

A final weakness of the present study, shared with all observational studies, is ambiguity concerning the causal relations linking early experience and later outcomes. Perhaps the results are due to a genetic, rather than experiential, factor that is shared by parent and child, which leads to both the observed differences in cortical thickness in adolescence and, in adulthood, to differences in the amount of environmental stimulation one is inclined to provide to children. Although common genetic influence on parentally provided home environment and child brain structure can explain an association between these measures, it cannot easily explain the age-dependence of the effect. That is, why would the parents’ genes influence the home environment they provide for the child at age 4 more than the home environment they provide for the child at age 8? Although logically conceivable, this account requires the assumption that certain genes influence both brain structure and adult parenting behaviors, the latter being child-age-dependent.

Should we expect estimates of heritability from studies of twins to dovetail with the present results, identifying complementary sets of areas? In principle yes, to a degree limited by different methods of cortical parcellation used across studies and, more importantly, the narrow sample of experience types and times measured here compared to the global and summated nature of experience measured in twin studies. Nevertheless, it is noteworthy that all of the areas identified in the present analysis fall among the less genetically determined half of those reviewed in Blokland et al.’s meta-analysis of cortical thickness studies [12]. More recent results, from Yang et al.’s study of 14 year-old twins [36], show low areas of heritability in the inferior lateral temporal areas found here to correlate with childhood environmental stimulation.

## Conclusion

In conclusion, the present results are the first to demonstrate a long-term and specific relationship between variation in normal childhood experience and brain structure later in life. They show that a specific aspect of childhood experience, measured at age 4, is predictive of cortical thickness in temporal regions in late adolescence.

## Supporting Information

S1 File Additional methodological details. (DOCX)

S2 File Results of alternative analysis with additional indices of ability co-varied. (DOCX)
