# Supplementary material for: Relation of Childhood Home Environment to Cortical Thickness in Late Adolescence: Specificity of Experience and Timing
Source: PLoS One. 2015 Oct 28;10(10):e0138217. doi: 10.1371/journal.pone.0138217 (PMC4624931; doi:10.1371/journal.pone.0138217)
Supplement: S1 File — (DOCX) [file pone.0138217.s001.docx]

**Supporting Information**

**Additional methodological details**

**Measures of childhood environment and covariates**

Two composites, measuring environmental stimulation and parental nurturance, were created by averaging the z-scores of the subscales listed here along with examples of items.

The *Environmental Stimulation* composite incorporated the following subscales for 4 year-olds (with two sample items from each): Learning stimulation (“child has toys which teach color,” “at least 10 books are visible in the apartment”), language stimulation (“child has toys that help teach the names of animals,” “mother uses correct grammar and pronunciation,”), academic stimulation (“child is encouraged to learn colors,” “child is encouraged to learn to read a few words”), modeling (“some delay of food gratification is expected,” “parent introduces visitor to child”), and variety of experience (“child has real or toy musical instrument,” “child’s art work is displayed some place in house”). For 8 year-olds, the relevant subscales were: Growth fostering materials and experiences (“child has free access to at least ten appropriate books,” ”house has at least two pictures of other type of art work on the walls”), provision for active stimulation (“family has a television, and it is used judiciously, not left on continuously,” “family member has taken child, or arranged for child to go to a scientific, historical or art museum within the past year”), family participation in developmentally stimulating experiences (“Family visits or receives visits from relatives or friends at least once every other week,” “family member has taken child, or arranged for child to go, on a trip of more than 50 miles from his home”).

The *Parental Nurturance* composite incorporated the following subscales for 4 year-olds (with two sample items from each): Warmth and affection (“parent holds child close 10-15 minutes per day,” “parent converses with child at least twice during visit”) and acceptance (“parent does not scold or derogate child more than once,” “parent neither slaps nor spanks child during visit”). For 8 year-olds, the subscales used were: Emotional and verbal responsivity (“Child has been praised at least twice during past week for doing something,” “parent responds to child’s questions during interview”), encouragement of maturity (“family requires child to carry out certain self care routines,” “parents set limits for child and generally enforce them”), emotional climate (“parent has not lost temper with child more than once during previous week,” “parent uses some term of endearment or some diminutive for child’s name when talking about child at least twice during visit”) and paternal involvement (“Father [or father substitute] regularly engages in outdoor recreation with child,” “Child eats at least one meal per day, on most days, with mother and father [or mother and father figure]”).

Concerning other variables with the potential to account for differences in neurocognitive development: Prenatal cocaine exposure was ascertained by maternal report and confirmed by maternal and infant urine test [15]. Maternal intelligence was measured by the Weschler Adult Intelligence Scale-Revised (WAIS-R) when the child was 6 years old. Participant IQ was measured by the WAIS-IV at age 18.

**Image analyses**

PipeDream automates and quality assures Advanced Normalization Tools processing (ANTs, <http://www.picsl.upenn.edu/ANTS/>) via a single parameter file and data organization hierarchy. Each subject’s T1 imaging data are inhomogeneity corrected via the N4 bias correction algorithm [37]. PipeDream then performs diffeomorphic normalization via the top-performing symmetric normalization methodology, available in ANTs, to map each subject to a population-specific template, built from the same scanner and imaging parameters. The template contains prior labeling and probability maps that are used to guide both brain extraction and neuroanatomical segmentation. Segmentation is performed with a Markov Random Field approach implemented in the ANTs tool Atropos, which has been validated on public datasets. Cortical thickness estimation is then performed with the DiReCT method [38].

The method of eigenanatomy, used here to derive meaningful sets of ROIs to examine for effects of the earlier childhood environment, is related to the familiar method of principal component analysis (PCA). Like PCA, it exploits covariation to identify a basis set (eigenvectors) that may be used to optimally reduce the dimensionality of the input data. It differs from PCA in that PCA eigenvectors for brain images may lack interpretability in that they are global in extent, i.e. involve voxels over the whole brain. Recent statistical research provides an alternative to traditional PCA that incorporates penalties on eigenvector values (usually `0 or `1 common in compressed sensing) that force the eigenvectors to be sparse [20-22]. Sparse eigenvectors are easier to interpret in biological application areas, such as neuroimaging and genetics, where the location along the eigenvector is associated with contextual information (such as an area of the brain). In the current work, eigenvectors that are sparse and have only non-negative values are termed eigenanatomy, highlighting the fact that the eigenvectors are localized and anatomically interpretable. The non-negativity constraint on eigenanatomy vectors allows them to be treated as weighted averages and employed in a statistical framework much like a traditional region of interest. Our recent presentation of a novel method for computing eigenanatomy is described in Refs 20-22.

Here, we employ eigenanatomy to gain a sparse non-negative matrix decomposition of cortical thickness measurements to generate biologically relevant descriptions of data in an unsupervised manner. Each sparse eigenanatomy component is associated with covarying anatomical structure and may be related statistically to other demographic measures such as cognition or disease status.

The sparseness parameters for eigenanatomy were also chosen to provide clustered eigenvectors that occupy less than 2% of the cortical surface. This parameter choice guarantees: (1) relatively few variables (50 predictors as opposed to the original number of voxels); (2) a nearly full parcellation of the cortex; (3) results that yield localization at the scale of major gyri and sulci. Details of this method are available online (http://www.picsl.upenn.edu/ANTS/) and the code is open-source and compatible with all major computing platforms.

**References cited in Supporting Information**

15. Hurt, H. *et al.* (1995) Cocaine-exposed children: follow-up through 30 months. *J. Dev. Behav. Pediatr.* **16,** 29–35.

19. Caldwell, B. M., & Bradley, R. H. (1984). *Home Observation for Measurement of the Environment (HOME)*. Little Rock, AR: University of Arkansas at Little Rock.

20. Avants, B. *et al.* in *Med. Image Comput. Comput.-Assist. Interv.* (2012) 206–213. at <http://link.springer.com/chapter/10.1007/978-3-642-33454-2_26>

21. Kandel, B. M., Wolk, D. A., Gee, J. C. & Avants, B. (2013) Predicting cognitive data from medical images using sparse linear regression. in *Inf. Process. Med. Imaging* 86–97 at <http://link.springer.com/chapter/10.1007/978-3-642-38868-2_8>

22. Dhillon, P., Wolk, D., Das, S., Ungar, L., Gee, J. & Avants, B. (2014) Subject-Specific Functional Parcellation via Prior Based Eigenanatomy. *NeuroImage*, 99, 14–27.

37. Tustison NJ, Avants BB, Cook PA, Zheng Y, Egan A, et al. (2010) N4itk: improved n3 bias correction. *IEEE Trans Med Imaging* **29**: 1310–1320.

38. Das, S. R., Avants, B. B., Grossman, M. & Gee, J. C. (2009) Registration based cortical thickness measurement. *NeuroImage* **45,** 867–879.
