# Supplementary material for: Relation of Childhood Home Environment to Cortical Thickness in Late Adolescence: Specificity of Experience and Timing
Source: PLoS One. 2015 Oct 28;10(10):e0138217. doi: 10.1371/journal.pone.0138217 (PMC4624931; doi:10.1371/journal.pone.0138217)
Supplement: S2 File — (DOCX) [file pone.0138217.s002.docx]

**Supporting Information 2**

**Results of alternative analysis with child verbal comprehension and perceptual reasoning abilities covaried in lieu of Full Scale IQ**

The substitution of two more specific IQ measures, the Verbal Comprehension Index (PCI) and the Perceptual Reasoning Index (PRI), for the single Full Scale IQ (FSIQ) measure of child ability led to the same conclusions as the original analysis. As with FSIQ, there was no relationship between either of these two more specific measures of ability and cortical thickness in any of the four areas discussed here. Also as with FSIQ, the presence of these ability measures in the model did not eliminate the effect of Environmental Stimulation. Other covariates were either significant or nonsignificant as before, with the exception that for the left lateral inferior posterior temporal area gestational cocaine exposure went from being significant at the 0.05 level to nonsignificant. Numerical values are shown here:

| **Eigenanatomy Region** | **Environmental Stimulation at Age 4** | **VCI** | **PRI** | **Other covariates**  * p<0.05  ** p<0.01  ***p<0.001 |
| --- | --- | --- | --- | --- |
| L lateral inferior  temporal - posterior | β = -0.398  p = 0.012 | β = 0.253  p = 0.129 | β = -0.024  p = 0.881 | GCE*, Age** |
| L lateral  inferior  temporal -anterior | β= -0.565  p = 0.001 | β = 0.082  p = 0.621 | β = -0.043  p = 0.795 | Age* |
| Bilateral fusiform | β = -0.370  p = 0.020 | β = 0.161  p = 0.334 | β = -0.098  p = 0.558 | Gender*** |
| R lateral inferior temporal | β = -0.384  p = 0.023 | β = -0.196  p = 0.267 | β = 0.132  p = 0.448 | Gender** |
